# The ADF4-RCAR12 module regulates ABA-mediated drought tolerance in *Arabidopsis*

**DOI:** 10.3389/fpls.2026.1930623

**Published:** 2026-09-03

**Authors:** Xiaoyi Li, Yihan Feng, Jiahan He, Yufei Li, Xufeng Li, Jianmei Wang, Yi Yang, Maohua Wang

**Affiliations:** 1 Key Laboratory of Bio-Resources and Eco-Environment of Ministry of Education, College of Life Sciences, Sichuan University, Chengdu, China; 2 Sichuan University-Hong Kong Polytechnic University Institute for Disaster Management and Reconstruction, Sichuan University, Chengdu, China

**Keywords:** ABA, ABA receptor, ADF4, *Arabidopsis*, drought stress

## Abstract

**Introduction:**

Drought is one of the most severe abiotic stresses limiting crop productivity, and abscisic acid (ABA)-induced stomatal closure is a key mechanism underlying plant drought resistance. Although ABA signaling has been extensively characterized in *Arabidopsis thaliana*, the role and regulatory mechanisms of microfilaments (F-actin) in this process remain poorly understood. Previously, we identified the actin-binding protein CAP1 as an interactor of the ABA receptor RCAR12 and a phosphorylation target of OPEN STOMATA 1 (OST1), suggesting a potential link between actin dynamics and ABA signaling.

**Methods:**

To investigate the role of Actin Depolymerizing Factor 4 (ADF4) in ABA signaling, we examined its interaction with RCAR12 using bimolecular fluorescence complementation (BiFC), GST pull-down, and co-immunoprecipitation (Co-IP) assays. Genetic interactions between ADF4 and RCAR12 were further analyzed using *ADF4*-overexpressing lines in the ABA receptor quadruple mutant 1124 (*pyr1 pyl1 pyl2 pyl4*) and the *adf4* × OE-*RCAR12* genotype. ABA responses and drought tolerance were evaluated through seed germination, cotyledon greening, stomatal closure, and drought assays.

**Results:**

ADF4 physically interacted with RCAR12 *in vivo* and *in vitro*. Overexpression of ADF4 only weakly rescued the ABA-insensitive phenotype of the 1124 quadruple mutant during seed germination and seedling establishment, but completely restored its drought sensitivity. In addition, the adf4 × OE-RCAR12 genotype partially rescued the defects of the adf4 mutant in ABA-mediated seed germination, cotyledon greening, ABA-induced stomatal closure, and drought tolerance.

**Discussion:**

These findings demonstrate that ADF4 functionally interacts with the ABA receptor RCAR12 and that the two proteins act synergistically in regulating ABA responses. Our results establish a link between actin dynamics and ABA receptor-mediated signaling and suggest that ADF4–RCAR12 coordination contributes to ABA-induced stomatal regulation and drought tolerance in Arabidopsis.

## Introduction

Abscisic acid (ABA) is a well-characterized phytohormone that plays a pivotal role in plant adaptation to drought and other abiotic stresses (Raghavendra et al., 2010; Wang et al., 2024). ABA receptors, known as Pyrabactin Resistance 1/PYR1-Like/Regulatory Component of ABA Receptor (PYR/PYL/RCAR, hereafter named RCAR), serve as key components of the ABA signaling pathway. Upon binding to ABA, RCARs interact with and inhibit type 2C protein phosphatases (PP2Cs), thereby releasing the inhibition of downstream signaling components and activating ABA signal transduction (Ma et al., 2009; Park et al., 2009). Previous studies have identified 14 members of the START/Bet v 1 superfamily in *Arabidopsis thaliana*, including PYRABACTIN RESISTANCE 1 (PYR1), PYR1-like (PYL) 1–13, also referred to as RCAR1–14 (Cutler et al., 2010). Based on their structural features and functional properties, these receptors are categorized into three subfamilies. Members of the first and second subfamilies primarily function as monomers, whereas those belonging to the third subfamily predominantly exist as dimers (Santiago et al., 2012).

Although the PYR/PYL/RCAR family exhibits extensive functional redundancy, accumulating evidence indicates that individual receptors possess distinct biological functions. ABA specifically stabilizes PYL8, promotes its accumulation in root nuclei, and facilitates its non-cell-autonomous movement between neighboring root tissues, providing a mechanistic basis for its unique roles in root development and environmental adaptation (Belda-Palazon et al., 2018). In contrast, RCAR7 (PYL13) represents an atypical member of the receptor family that does not act as a canonical ABA receptor (Zhao et al., 2013). Instead, RCAR7 (PYL13) modulates ABA signaling through ABA-independent interactions with PP2Cs and other PYL proteins, highlighting the functional diversification among ABA receptors. Moreover, the expression of ABA receptors is tightly regulated at the transcriptional level. The transcription factor ABI5 directly regulates the expression of multiple PYL genes, particularly PYL11 and PYL12, thereby establishing a feedback regulatory mechanism that fine-tunes ABA-mediated inhibition of seed germination (Zhao et al., 2020).

More recently, post-translational regulation of ABA receptors has emerged as an important area of investigation. The abundance and activity of ABA receptors are tightly controlled through ubiquitination and endomembrane trafficking pathways (Yang et al., 2017). For example, the RSL1–FYVE1-mediated endocytic pathway promotes the vacuolar degradation of PYL4 and related receptors, thereby attenuating ABA signaling (Belda-Palazon et al., 2016). A DWD protein, AtRAE1 (RNA export factor 1 in Arabidopsis), which may serve as a substrate receptor of the CUL4–DDB1 E3 ubiquitin ligase complex, was reported to interact with RCAR1/PYL9, suggesting a potential role for ubiquitin-mediated regulation in ABA receptor homeostasis (Li et al., 2017). Beyond ubiquitination, ABA receptors are also regulated by phosphorylation mediated by protein kinases, which modulates their stability, activity, or signaling capacity. RCAR3 and RCAR11 are phosphorylated by cytosolic ABA receptor kinases, thereby promoting their interaction with ABA (Li et al., 2019, 2022a; Zhang et al., 2018). The TOR kinase phosphorylates ABA receptors to suppress stress responses under favorable growth conditions and facilitate growth recovery after the cessation of environmental stresses (Wang et al., 2018). In addition, rice OsPYL/RCAR5 and OsPYL/RCAR7, as well as BdPYL1 from Brachypodium distachyon, have been shown to positively regulate ABA responses and enhance abiotic stress tolerance (Bhatnagar et al., 2020; Kim et al., 2012; Pri-Tal et al., 2017). Collectively, research on ABA receptors has progressed from establishing the core signaling framework to elucidating receptor-specific functions, regulatory mechanisms underlying protein turnover, and potential applications in crop improvement, providing important insights into the molecular basis of plant stress adaptation.

Stomatal regulation is a critical adaptive strategy that enables plants to reduce water loss under drought conditions (Rodriguez et al., 2019). Accumulating evidence indicates that the actin cytoskeleton plays an essential role in controlling stomatal movement, while actin-binding proteins (ABPs) coordinate the dynamic remodeling of actin filaments at multiple levels (Jiang et al., 2012; Li et al., 2022b; Yang et al., 2022). Among these regulators, Actin Depolymerizing Factor/cofilin (ADF/cofilin) proteins are evolutionarily conserved modulators of actin dynamics that control actin filament organization through their severing and depolymerization activities (Inada, 2017). In Arabidopsis thaliana, the ADF family consists of 11 members that are grouped into four subfamilies, with ADF4 belonging to the first subfamily and displaying relatively high expression levels (Ruzicka et al., 2007). The expression of Arabidopsis *ADF8* and *ADF11* is regulated by the transcription factor RSL4, auxin-responsive ARF proteins, and the ethylene-responsive transcription factor EIN3, indicating that hormonal cues modulate F-actin dynamics through transcriptional regulation of ADF8/11 (Li et al., 2026a). Previous studies have shown that casein kinase 1-like protein 2 (CKL2) phosphorylates ADF4 and regulates stomatal closure during abiotic stress responses in *A. thaliana* (Zhao et al., 2016). Moreover, ADF4 has been proposed to participate in ABA signaling through the PYR–PP2C–CKL2 regulatory module (Shi et al., 2022). Unlike ADF4, ADF7 and ADF10 are mainly associated with reproductive and developmental processes, including pollen tube elongation and root hair development (Daher and Geitmann, 2012; Li et al., 2024; Qu et al., 2022; Wang et al., 2023). However, the molecular relationship between ABA receptors and ADF4 remains largely unknown.

In this study, we demonstrate that the ABA receptor RCAR12 interacts with the actin-depolymerizing factor ADF4 in plant cells. Phenotypic analyses revealed that RCAR12 positively regulates ABA-mediated drought tolerance through its association with ADF4.

## Materials and methods

### Plant materials and growth conditions

Plant materials and growth conditions Arabidopsis plants used in this study were all in the Columbia (Col-0) background. The *adf4* T-DNA insertion mutants, OE-*ADF4* #10 overexpression plants were previously described (Peng et al., 2022b; Yao et al., 2022). Plants were grown on soil-vermiculite mixtures at 22 °C under 60% relative humidity with cycles of 16 h light and 8 h dark. For plate experiment, seeds were stratified in distilled water for 3 days at 4 °C in dark. The imbibed seeds were surface sterilized with a 20% bleach solution for 15 min and then washed 5 times with sterilized water, and then sown on Murashige and Skoog (MS) medium containing 2% sucrose and 1.0% agar, pH 5.8.

### Plasmid construction and generation of transgenic plants

To generate the cLUC-ADF4 construct, the full-length coding sequence of ADF4 was cloned into the *Bam*HI-*Sal*I sites of the *p*Cambia1300-cLUC vector. To generate *p*EarleyGate100-RCAR12-GFP, the full-length coding sequence of RCAR12 was cloned into the *Xho*I site of the *p*EarleyGate100 vector. The primers are shown in **Supplementary Table 1**.

To test the interaction of RCAR12 with ADF4 in ABA signaling, we obtained the *adf4* × OE-*RCAR12* by crossing. For the 1124: OE-*ADF4* genotype, *p*Cambia1307-ADF4-4MYC construct was previously described (Yao et al., 2022) and transformed into Agrobacteria GV3101 stain. And then transformed into the 1124 (loss-of-function of *PYR1*, *PYL1*, *PYL2* and *PYL4*, namely *RCAR11*, *RCAR12*, *RCAR14* and *RCAR10*) mutants according to floral dip method. Positive lines were screened with hygromycin, and homozygotes were harvested in the T3 generation for phenotype analysis. The mRNA and protein levels were identified by RT-qPCR and immunoblotting, respectively. For overexpressing-*RCAR12* plants, the *p*EarleyGate100-RCAR12-GFP construct was transformed into Agrobacteria GV3101 stain and infiltrated into the Col-0 plants. Positive line was screened with Basta and determined by immunoblotting using anti-GFP.

### Yeast two-hybrid assays

The two-hybrid assays were performed as described previously (Li et al., 2026b; Yao et al., 2022). Briefly, the indicated combinations of BD-RCARs and AD-ADF4, AD-CARK3, or empty vector were cotransformed into the yeast (*Saccharomyces cerevisiae*) strain AH109, respectively. The yeast transformants were selected on the SD/-Leu (L)/-Trp (W) agar plates and then grown on the SD/-Leu (L)/-Trp (W)/-His (H)/-Ade (A) agar plates. Photos were taken after incubation at 30 °C for 2–4 d.

### Bimolecular fluorescence complementation assay

The ADF4-YFP^N^, ABI1-YFP^N^ and RCARs-YFP^C^ constructs were previously described (Li, et al., 2022a, Peng, et al., 2022b, Zhang et al., 2018). The constructs were transformed into Agrobacteria GV3101 stain. And then the indicated YFP^N^/YFP^C^ combination were co-expressed in 4-week old tobacco leaves. After 2 days, the signals of YFP were observed with a Leica confocal laser scanning microscope (DM4 B). 3 leaves were observed each time and three biological repeats were performed.

### GST pull-down assay

To test the interaction of RCAR12 with ADF4 *in vitro*, GST-RCAR12, GST and His-ADF4 were purified as previously described (Li et al., 2019; Yao et al., 2022). 5 μg GST-RCAR12 or GST bound glutathione-agarose beads were incubated with His-ADF4 in binding buffer (50 mM Tris-HCl, pH 8.0, 200 mM NaCl, 10% glycerol, 0.1% Tween 20) at room temperature for 30 min and washed 10 times with binding buffer. The pulled-down compounds were eluted with 2× SDS sample buffer (25 mM Tris-HCl, pH 6.8, 10% glycerol, 0.8% SDS, and 2% 2-mercaptoethanol) and boiled at 98 °C for 10 min. Next, proteins were analyzed by immunoblotting with GST and His antibodies.

### Split‐LUC complementation analysis

For split LUC complementation analysis, RCAR12-nLUC was cotransformed with cLUC-ADF4 into *N. benthamiana* leaves by *Agrobacterium tumefaciens*-mediated transformation. The infiltrated plants were grown overnight and under long‐day conditions for 2 d and then subjected for LUC signal detection to determine whether the two proteins interacted with each other.

### Statistical analysis

In this study, statistical data were analyzed using GraphPad Prism version 8 software (GraphPad Software, La Jolla, CA, USA). Data were collected in triplicates and subjected to analysis of variance (ANOVA). *Post hoc* means separation was done with Tukey’s test at *P* < 0.05.

### Physiological analysis

For ABA-mediated germination, 0, 0.5, 1, 3 or 5 μM ABA was added to half-strength MS medium for 5 or 7 days. Germination and cotyledon greening were performed as previously described (Li et al., 2022a). For germination assays, seeds were plated on MS medium without or with ABA as indicated in the text and figure legends and were then stratified at 4 °C for 3 d. The rates of seed germination, cotyledon greening were scored after the indicated time intervals. Seedlings were grown vertically for 4d before transferring to medium without or with the indicated concentrations of ABA; root phenotypes were evaluated at 9 d after transferring.

For drought tolerance experiment, 7-d-old seedlings indicated genotypes were grown under the same conditions with sufficient watering and then subjected to drought stress treatment by withholding water for 12 or 14 days. Photos were taken after drought treatment. Then, 2 days after rehydration, the plants were photographed, and the survival rate was then recorded.

Stomatal aperture was carried out as previously described (Li, et al., 2022a). Three-week old leaves were peeled and then incubated in opening buffer (50 mM KCl, 10 μM CaCl_2_, 10 mM MES pH 6.15) for 3 h to induce stomatal opening. For ABA treatment, 20 μM ABA was added to the opening buffer for 3 h. Images were taken using Leica TCS SP5 II HCS confocal microscope.

## Results

### ADF4 physically interacts with the ABA receptor RCAR12

To investigate whether ABA receptors interact with ADF4, we performed yeast two-hybrid (Y2H) assays. The results showed that ADF4 interacted with multiple ABA receptors, including RCAR2, RCAR3, RCAR6, RCAR7, RCAR8, RCAR10, RCAR12, and RCAR13 (**Supplementary Figure 1**). We further examined these interactions in tobacco cells using bimolecular fluorescence complementation (BiFC) assays. YFP fluorescence signals were detected when ADF4 was co-expressed with RCAR3, RCAR7, RCAR8, or RCAR12, whereas no fluorescence was observed in the corresponding negative controls (**Supplementary Figure 2**). Based on these results, RCAR12 was selected for further investigation.

**Figure 1:**
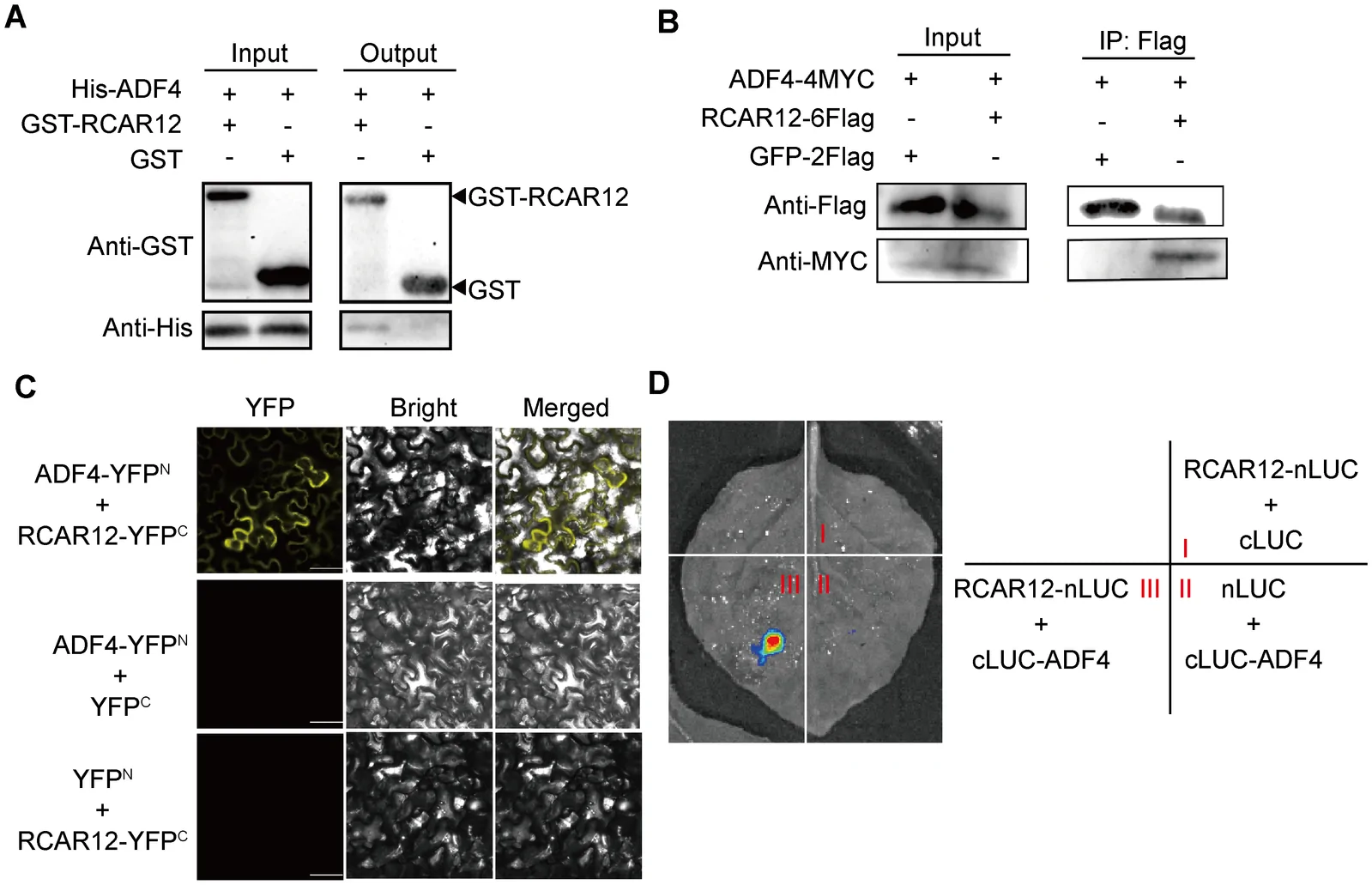
ADF4 interacts with RCAR12. **(A)** ADF4 interacts with RCAR12 in GST pull-down assay. His-ADF4 was incubated with immobilized GST–RCAR12 or GST. Proteins retained on the Glutathione Sepharose after washing were eluted and probed using anti-His and anti-GST antibodies. **(B)** The interaction of ADF4 with RCAR12 was tested using a co-immunoprecipitation (Co-IP) assay. Total proteins were extracted from *Nicotiana benthamiana* leaves coexpressing ADF4-4MYC and RCAR12-6Flag, or GFP-2Flag and immunoprecipitation was carried out with anti-Flag reagent for 4 h at 4 °C. Immunoblot assay was detected with anti-Flag and anti-MYC antibodies. **(C)** Analysis of the interaction between ADF4 protein and RCAR12 protein was determined by bimolecular fluorescence complementation (BiFC) assay. ADF4 and RCAR12 were coexpressed in epidermal cells of *N. benthamiana*. Scale bars = 30 μm. **(D)** ADF4 interacts with RCAR12 in split-Luc complementation assay. Indicated constructs were transiently expressed in one single leaf of *N. benthamiana*. LUC signals were analyzed at 2 d after infiltration.

We first performed an *in vitro* GST pull-down assay to verify the direct interaction between ADF4 and RCAR12. As shown in **Figure 1A**, His-ADF4 was specifically pulled down by GST-RCAR12, but not by GST alone, demonstrating a direct physical interaction between ADF4 and RCAR12 *in vitro*. To further confirm this interaction in planta, co-immunoprecipitation (Co-IP) assays were performed in *N. benthamiana* leaves. ADF4-4MYC was efficiently co-immunoprecipitated with GFP-RCAR12-Flag using anti-Flag beads, whereas no detectable signal was observed in the control samples (**Figure 1B**), confirming their association in plant cells. The interaction between ADF4 and RCAR12 was further validated using BiFC and split luciferase complementation assays. Strong YFP fluorescence was detected in tobacco cells co-expressing ADF4-YFPN and RCAR12-YFPC, whereas no fluorescence signal was observed in negative controls expressing either fusion protein with the corresponding empty vector (**Figure 1C**). Consistently, co-expression of RCAR12-nLUC and cLUC-ADF4 generated a strong luminescence signal, while control combinations exhibited negligible luciferase activity (**Figure 1D**). Collectively, these results demonstrate that the ABA receptor RCAR12 physically interacts with ADF4 both *in vitro* and in planta, suggesting a potential functional connection between ABA perception and ADF4-mediated regulation of actin dynamics.

### ADF4 and ABA receptors are involved in ABA signaling pathway and drought tolerance

**Figure 2:**
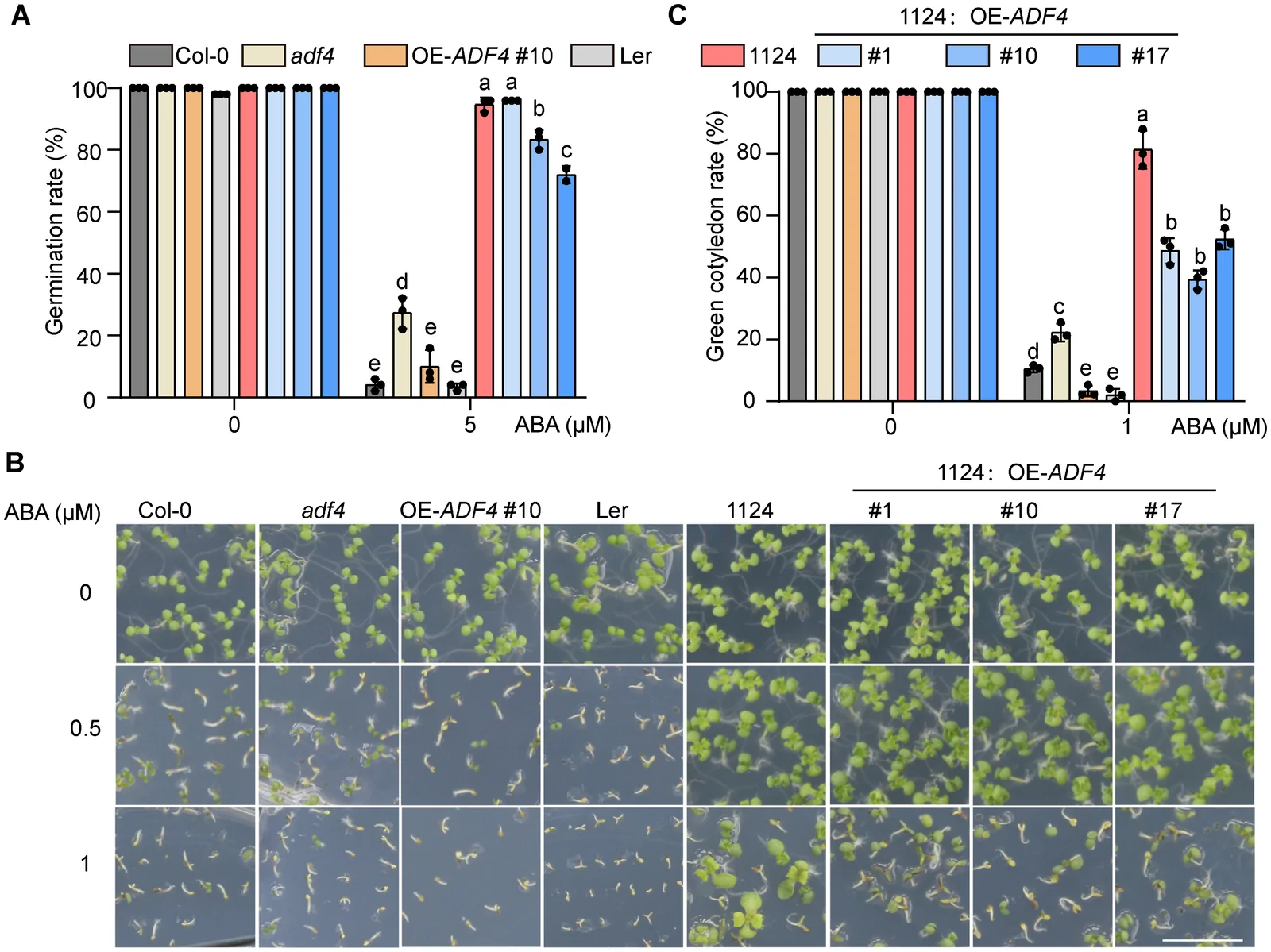
Overexpression of *ADF4* partially recovers the hypersensitive to ABA in 1124 mutants. **(A)** Germination rate measurement. Imbibed WT (Col-0 and Ler), *adf4*, OE-*ADF4* #10 and 1124: OE-*ADF4* (#1/#10/#17lines) seeds were sown on 1/2 MS medium containing 0 or 5 μM ABA and then seed germination rates after 7 days were calculated. Germination was defined as the first sign of radicle tip emergence (n = 3, each pool containing 50 seeds). Different letters represent significant differences determined by one-way ANOVA with Tukey’s *post hoc* test (*P* < 0.05). **(B)** The phenotype of Arabidopsis seedlings in response to ABA treatment. Seeds were grown vertically on 1/2 MS medium containing 0, 0.5 or 1 μM ABA for 7 days. Scale bar = 1 cm. **(C)** Seedling establishment rate measurements in **(B)**. (n = 3, each pool containing 50 seedlings). Different letters represent significant differences determined by one-way ANOVA with Tukey’s *post hoc* test (*P* < 0.05). The rates of seedling establishment were measured based on the criteria recently used by previous studies that hypocotyls and cotyledons should emerge completely in established seedlings (Ma et al., 2023; Peng et al., 2022a).

To determine whether ADF4 is involved in ABA-mediated inhibition of early seedling development, we examined seed germination and cotyledon greening in wild-type, *adf4*, *ADF4*-overexpressing, and ABA receptor mutant plants. Under control conditions, all genotypes exhibited comparable germination rates and cotyledon greening, indicating that ADF4 has little effect on early seedling establishment in the absence of ABA (**Figures 2A–C**). In the presence of 5 μM ABA, seed germination of Col-0 was markedly inhibited, whereas the *adf4* mutant displayed a significantly higher germination rate than Col-0 (**Figure 2A**). In contrast, *ADF4*-overexpressing plants did not exhibit enhanced ABA sensitivity compared with the wild type. These results indicate that loss of *ADF4* reduces ABA sensitivity during seed germination.

To further investigate the genetic relationship between ADF4 and ABA receptors, we introduced the *ADF4* overexpression construct into the 1124 mutant background (Peng et al., 2022b), which carries mutations in four ABA receptor genes (p*yr1 pyl1 pyl2 pyl4*), and examined its response to ABA during seed germination and early seedling development. As reported previously (Park et al., 2009), the 1124 mutant exhibited reduced sensitivity to ABA during seed germination and cotyledon greening. To investigate the genetic relationship between ADF4 and ABA receptors, we generated three independent *ADF4*-overexpressing lines in the 1124 background (**Supplementary Figures 3A, B**). Notably, *ADF4* overexpression significantly decreased both seed germination and cotyledon greening rates in the 1124 background compared with the parental mutant, although the transgenic lines remained significantly less sensitive to ABA than wild-type plants (**Figures 2A–C**). These results indicate that *ADF4* overexpression partially suppresses the ABA-insensitive phenotype of the 1124 mutant, suggesting that ADF4 functions, at least in part, downstream of or in coordination with ABA receptor-mediated signaling during early seedling development.

**Figure 3:**
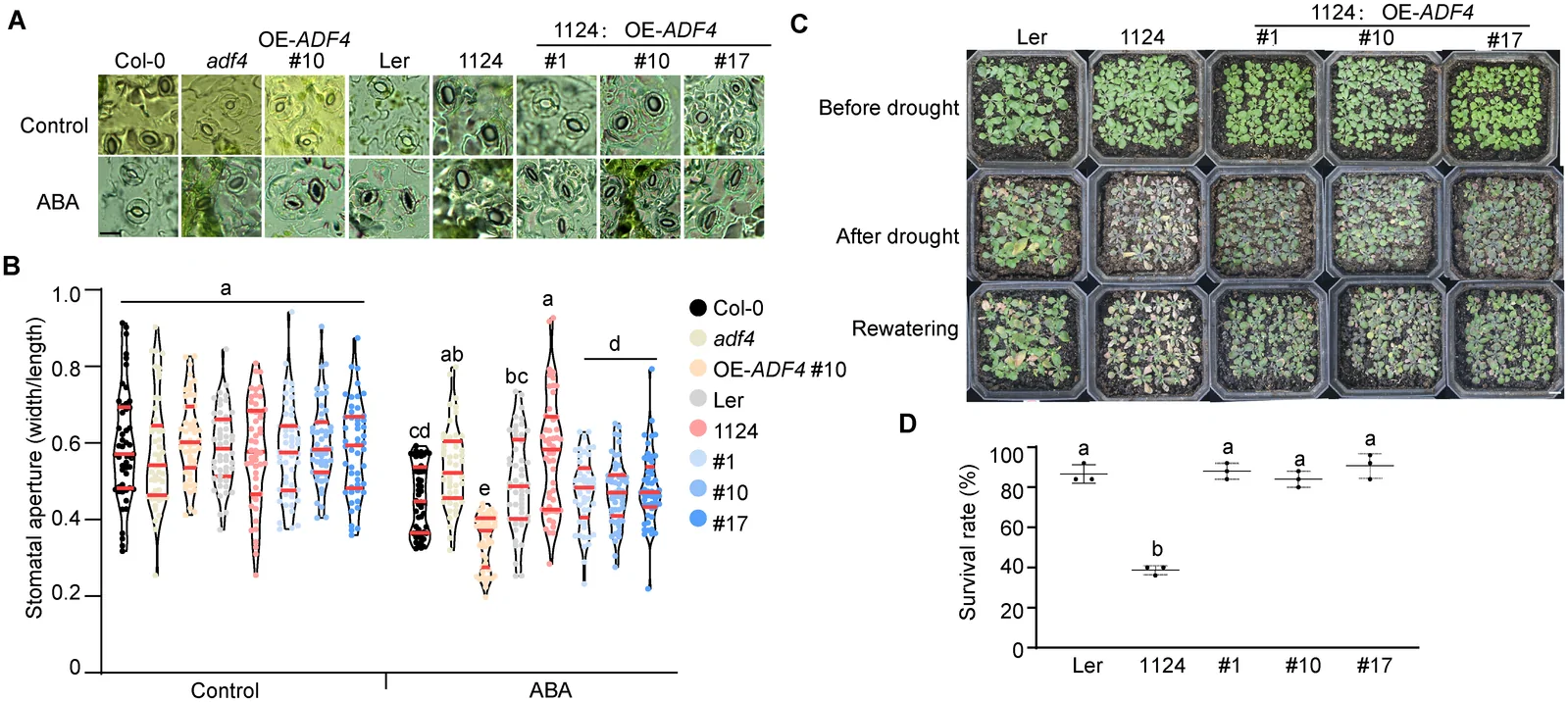
Overexpression of *ADF4* recovers the hypersensitive to drought tolerance in 1124 mutants. **(A)** Representative images of stomata in wild-type (Col-0 and Ler), *adf4*, OE-*ADF4* #10 and 1124: OE-*ADF4* with or without 20 mM ABA for 3 h. Scale bar = 20 μm. **(B)** Quantification of stomatal closure in different genotypes shown in **(A)**. Values shown are means ± SD (n = 50), with 10 plants analyzed. Different lowercase letters represent significant differences, as determined by two-way ANOVA in combination with Tukey’s multiple comparisons test (*P* < 0.05). **(C)** Morphology of seedlings before and after drought stress treatment. Two-week-old wild-type, 1124, and 1124: OE-*ADF4* plants were subjected to drought stress for 13 days and then rewatered for 2 days. Scale bar = 1 cm. **(D)** Survival rate after drought treatment. Values shown are means ± SD (n = 3 biological replicates). Different lowercase letters represent significant differences, as determined by one-way ANOVA in combination with Tukey’s multiple comparisons test (*P* < 0.05).

Next, we examined ABA-induced stomatal responses and drought tolerance in the different genotypes. Under control conditions, stomatal apertures were comparable among all genotypes (**Figures 3A, B**). Upon ABA treatment, stomata of wild-type plants closed markedly. In contrast, *ADF4*-overexpressing plants exhibited significantly smaller stomatal apertures than the wild-type plants, indicating enhanced sensitivity to ABA (**Figures 3A, B**). Notably, overexpression of *ADF4* in the 1124 background partially restored the impaired ABA-induced stomatal closure of the 1124 mutant, although the transgenic plants remained less responsive than the wild-type plants (**Figures 3A, B**). To further examine the genetic relationship between ADF4 and ABA receptors in drought responses, plants were subjected to a drought treatment followed by rewatering. As expected, the 1124 mutant exhibited markedly reduced drought tolerance, as evidenced by severe wilting and a low survival rate after rewatering. In contrast, *ADF4*-overexpressing lines in the 1124 background exhibited drought tolerance comparable to that of wild-type plants, with a significantly increased survival rate relative to the 1124 mutant (**Figures 3C, D**). These findings indicate that *ADF4* overexpression is sufficient to rescue the drought-sensitive phenotype caused by the loss of multiple ABA receptors, suggesting that ADF4-mediated drought tolerance is largely independent of canonical ABA receptor signaling.

### RCAR12 mediates ABA responses partially through ADF4

To further investigate the genetic relationship between ADF4 and RCAR12, we generated *RCAR12*-overexpressing plants in the Col-0 background (OE-*RCAR12*). The *adf4* × OE-*RCAR12* plants were obtained by genetic crossing, and RCAR12 protein accumulation and *RCAR12* transcripts were confirmed (**Supplementary Figures 4A–C**). ABA responses of the different genotypes were evaluated during seed germination, cotyledon greening, stomatal movement, and drought tolerance.

**Figure 4:**
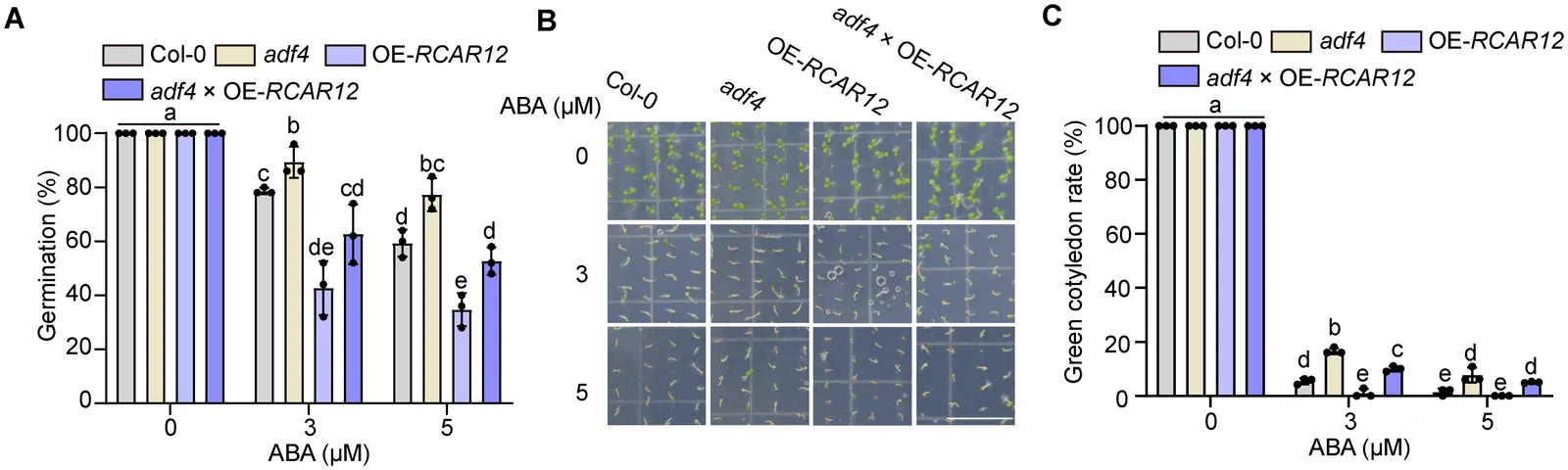
ADF4 is required for RCAR12 involved in ABA signaling. **(A)** Germination rate measurement. Imbibed WT (Col-0) seeds were sown on 1/2 MS medium containing 0, 3 or 5 μM ABA and then seed germination rates after 4 days were calculated. Germination was defined as the first sign of radicle tip emergence (n = 3, each pool containing 50 seeds). Different letters represent significant differences determined by one-way ANOVA with Tukey’s *post hoc* test (*P* < 0.05). **(B)** The phenotype of Arabidopsis seedlings in response to ABA treatment. Seeds were grown vertically on 1/2 MS medium containing 0, 3 or 5 μM ABA for 7 days. Scale bar = 1 cm. **(C)** Seedling establishment rate measurements after 7–10 days. (n = 3, each pool containing 50 seedlings). Different letters represent significant differences determined by one-way ANOVA with Tukey’s *post hoc* test (*P* < 0.05).

**Figure 5:**
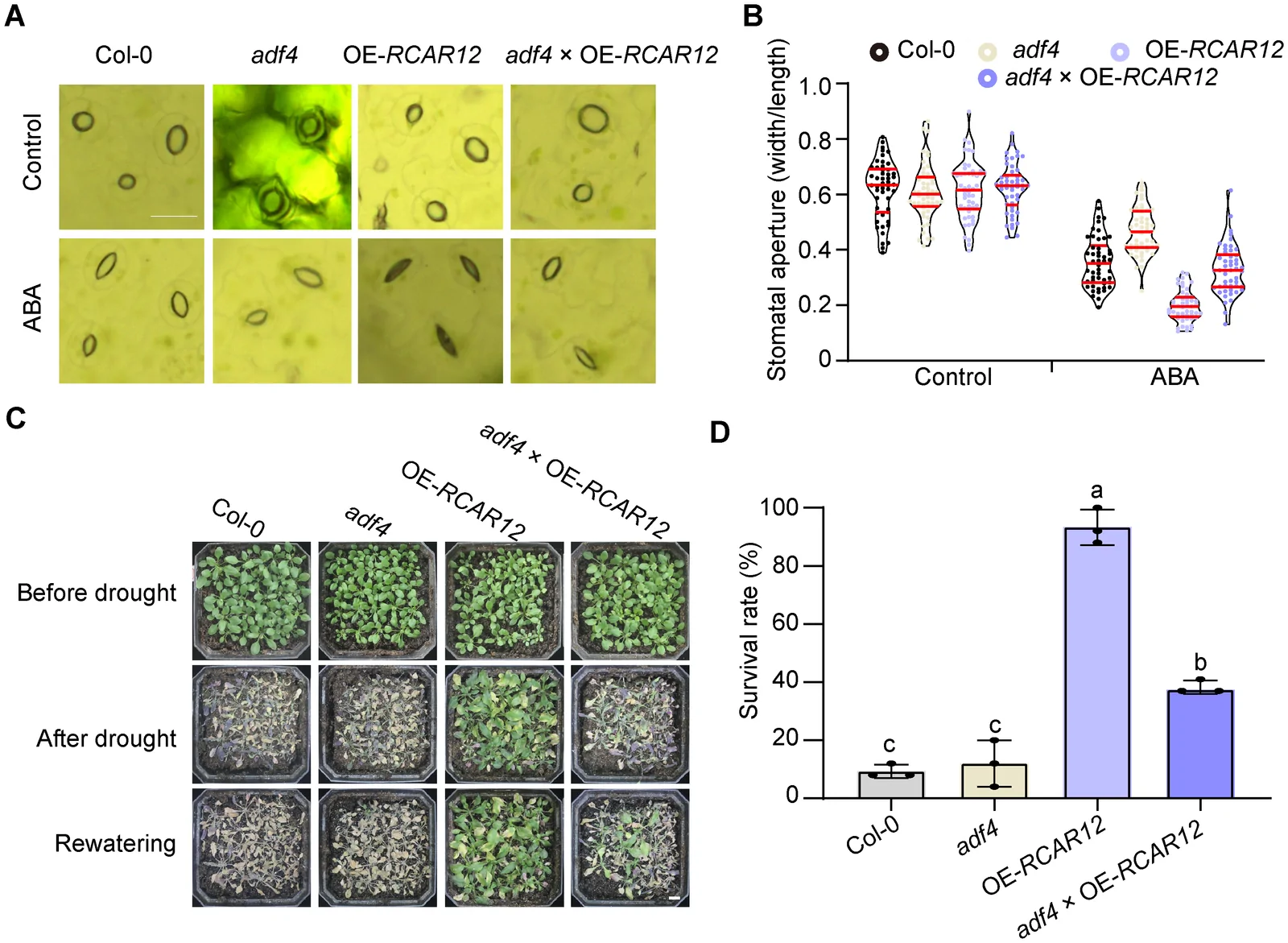
ADF4 is required for RCAR12 in response to ABA-mediated drought tolerance in Arabidopsis. **(A)** Representative images of stomata in wild-type (Col-0), *adf4*, OE-*RCAR12* and *adf4* × OE-*RCAR12* with or without 20 mM ABA for 3 h. Scale bar = 20 μm. **(B)** Quantification of stomatal closure in different genotypes shown in **(A)**. Values shown are means ± SD (n = 50), with 10 plants analyzed. Different lowercase letters represent significant differences, as determined by two-way ANOVA in combination with Tukey’s multiple comparisons test (*P* < 0.05). **(C)** Morphology of seedlings before and after drought stress treatment. 2-week-old wild-type, *adf4*, OE-*RCAR12* and *adf4* × OE-*RCAR12* plants were subjected to drought stress for 14 days and then rewatered for 2 days. Scale bar = 1 cm. **(D)** Survival rate after drought treatment. Values shown are means ± SD (n = 3 biological replicates). Different lowercase letters represent significant differences, as determined by one-way ANOVA in combination with Tukey’s multiple comparisons test (*P* < 0.05).

Under control conditions, no significant differences in seed germination or cotyledon greening were observed among the genotypes. Overexpression of *RCAR12* in the *adf4* background partially restored the ABA-hypersensitive germination and cotyledon greening phenotypes observed in OE-*RCAR12* plants, although the response was weaker than that of the OE-*RCAR12* line (**Figures 4A–C**). A similar genetic relationship was observed for stomatal responses and drought tolerance. Upon ABA treatment, *adf4* × OE-*RCAR12* plants displayed smaller stomatal apertures than the *adf4* mutant, no significant difference with Col-0, but larger apertures than OE-*RCAR12* plants (**Figures 5A, B**). Consistent with the stomatal phenotype, *adf4* × OE-*RCAR12* plants exhibited substantially enhanced drought tolerance relative to the *adf4* mutant and Col-0, although their survival rate remained lower than that of OE-*RCAR12* plants (**Figures 5C, D**). These genetic data indicate that ADF4 is partially required for RCAR12-mediated ABA responses and drought tolerance.

## Discussion

Reduced water availability induces ABA accumulation, which is perceived by members of the PYR/PYL/RCAR receptor family and activates signaling responses essential for plant adaptation to drought stress, as well as for the regulation of growth and development (Chen et al., 2022; Zhao et al., 2018). Previous studies suggested that ADF4 participates in ABA signaling through its interaction with the casein kinase-like protein CKL2 (Zhao et al., 2016). CKL2 kinase activity is inhibited by the clade A PP2C phosphatases ABI1 and ABI2 (Shi et al., 2022). Under drought conditions, ABA signaling releases CKL2 from PP2C-mediated inhibition, allowing activated CKL2 to phosphorylate ABI1 and thereby stabilize ABI1 protein. However, how ABA receptors functionally coordinate with F-actin-associated proteins to regulate stomatal movement under drought stress remains poorly understood.

In this study, we demonstrate that the ABA receptor RCAR12 interacts with ADF4 and that this interaction contributes to ABA signaling, including seed germination, seedling establishment, stomatal closure, and drought tolerance. Overexpression of *ADF4* partially rescued the ABA-insensitive phenotype of the 1124 receptor mutant during germination and stomatal responses, whereas overexpression of *RCAR12* substantially restored ABA responses and drought tolerance in the adf4 mutant background (**Figures 2**-**5**). Our results further suggest that ADF4-mediated regulation of actin dynamics is closely associated with ABA-induced stomatal closure. *ADF4*-overexpressing plants exhibited enhanced ABA-induced stomatal closure, whereas the adf4 mutant displayed reduced ABA sensitivity in stomatal responses (**Figures 3A, B**). Consistent with our findings, loss-of-function mutations in *ADF5* resulted in increased water loss from detached leaves, reduced survival rates following drought stress, and delayed stomatal closure (Qian et al., 2019), highlighting the conserved roles of ADF/cofilin proteins in regulating stomatal function and drought responses. These observations are also consistent with previous findings showing that CARK3 phosphorylates ADF4 to regulate ABA-dependent drought tolerance (Peng et al., 2022b). Notably, overexpression of *ADF4* in the 1124 background substantially rescued the drought-sensitive phenotype caused by the loss of multiple ABA receptors, suggesting that ADF4 may promote drought tolerance through both ABA receptor-dependent and partially receptor-independent mechanisms. However, because ADF4 overexpression did not fully restore ABA sensitivity and *ADF4*-overexpressing plants displayed reduced ABA responsiveness in stomatal regulation, ADF4 likely represents one component of a broader regulatory network controlling ABA responses rather than a core ABA signaling component. These findings support a genetic and functional relationship between RCAR12 and ADF4 in ABA-mediated stress responses.

A possible model is that, under drought stress, ABA perception activates RCAR12 and promotes its association with ADF4, thereby linking ABA receptor signaling to F-actin remodeling. Previous work showed that RCAR12 is released from CAP1 under drought stress (Li et al., 2026b). Activated RCAR12 may then interact with ADF4 to promote actin filament turnover. Meanwhile, CAP1 is phosphorylated by OST1 and inhibits microfilament disassembly, which may help maintain stomatal closure (Li et al., 2026b). In parallel, CKL2 is activated by ABA signaling and phosphorylates ABI1, thereby modulating ABA responses (Shi et al., 2022). CKL2 has also been reported to phosphorylate ADF4 and inhibit its actin-depolymerizing activity, contributing to the regulation of stomatal closure (Zhao et al., 2016). Together with our previous finding that ADF4 is phosphorylated by CARK3 to regulate drought tolerance, these results suggest that ABA signaling regulates stomatal movement through multiple converging kinase- and receptor-mediated pathways that ultimately target actin cytoskeleton dynamics.

In addition to RCAR12, our interaction assays showed that ADF4 also interacts with RCAR7/PYL13 (**Supplementary Figures 1**, **2**). RCAR7/PYL13 is an atypical member of the ABA receptor family and is largely insensitive to ABA (Li et al., 2013). Previous studies reported that RCAR7/PYL13 can promote drought tolerance through ABA-independent inhibition of PP2CA (Zhao et al., 2013). Moreover, PYL13 has been suggested to antagonize ABA-responsive PYLs by reducing their ABA-binding capacity. Therefore, it is possible that ADF4 may cooperate with different ABA receptor family members to regulate drought tolerance through both ABA-dependent and ABA-independent mechanisms, potentially via modulation of microfilament dynamics. Interestingly, our previous *in vitro* data showed that RCAR12 does not directly affect the actin-depolymerizing activity of ADF4 (Li et al., 2026b). Under high concentrations of exogenous ABA, 1124: OE-*ADF4* plants remained less sensitive to ABA during seed germination and cotyledon greening than *adf4* × OE-*RCAR12* plants (**Figures 2A–C**). This genetic relationship suggests that ADF4 may function in coordination with, rather than strictly downstream of RCAR12 to fine-tune ABA responses. For example, it is possible that ADF4 modulates the ABA-binding activity of RCAR12. Therefore, further studies are required to elucidate how the ADF4–RCAR12 interaction regulates actin dynamics and ABA-mediated drought responses in Arabidopsis.

In summary, our results demonstrate that ADF4 interacts with ABA receptors and participates in ABA signaling, ABA-induced stomatal closure, and drought tolerance in Arabidopsis. These findings provide new insight into the functional connection between ABA perception and actin cytoskeleton remodeling during plant drought adaptation.

## Data Availability

The raw data supporting the conclusions of this article will be made available by the authors, without undue reservation.
