# Supplementary material for: The ADF4-RCAR12 module regulates ABA-mediated drought tolerance in Arabidopsis
Source: Front Plant Sci. 2026 Sep 3;17:1930623. doi: 10.3389/fpls.2026.1930623 (PMC13581890; doi:10.3389/fpls.2026.1930623)
Supplement: Supplementary file 1 [file Supplementaryfile1.docx]

Supplementary Figure 1 Yeast-two-hybrid assays showing interactions between the RCAR family and ADF4. Yeast cells grown on an SD/-Leu (L)/-Trp (W) medium are shown as transform controls. Protein interaction is indicated by yeast cells growing well on the SD/-Leu (L)/-Trp (W)/-His (H)/-Ade (A) medium. Dilutions (0.1, 0.01, and 0.001) of yeast cells were spotted on the plates. Photographs were taken after 2 and 4 days. AD, pGADT7 vector; BD, pGBKT7 vector. The interaction of CARK3 and RCAR12 was set as a positive control.

Supplementary Figure 2 **Bimolecular Fluorescence Complementation (BiFC)** assays showing interactions between the RCAR family and ADF4. The interaction of ABI1 and RCAR3 was set as a positive control.

Supplementary Figure 3 The identification of OE-*ADF4*: 1124 plants. (A) The *ADF4* expression in 1124, 1124: OE-*ADF4* #1/#10/#17 lines. The results represent the means ± SD (n = 3). One-way ANOVA was carried out to determine the significance of difference (**P* < 0.05; ****P* < 0.001). (B) The ADF4 protein levels in plants. The total proteins were extracted and tested by immublotting using Anti-MYC. The loading was determined using Coomassie Brilliant Blue (CBB).

Supplementary Figure 4 The identification of *adf4* x OE-*RCAR12* plants. (A) The identification of the *adf4* T-DNA mutant. (B) The RCAR12 protein levels in plants. The total proteins were extracted and tested by immublotting using Anti-GFP. The loading was determined using Coomassie Brilliant Blue (CBB). (C) The *RCAR12* expression in Col-0, *adf4*, OE-*RCAR12* and *adf4* x OE-*RCAR12* seedlings determined by RT-qPCR. The results represent the means ± SD (n = 3). Different letters represent significant differences determined by one-way ANOVA with Tukey’s post hoc test (*P* < 0.05).
